# Developmental Origins of Cardiometabolic Diseases: Role of the Maternal Diet

**DOI:** 10.3389/fphys.2016.00504

**Published:** 2016-11-16

**Authors:** João H. Costa-Silva, Aiany C. Simões-Alves, Mariana P. Fernandes

**Affiliations:** Departamento de Educação Física e Ciências do Esporte, Centro Acadêmico de Vitória, Universidade Federal de PernambucoPernambuco, Brazil

**Keywords:** developmental plasticity, perinatal nutrition, cardiometabolic control, protein restriction

## Abstract

Developmental origins of cardiometabolic diseases have been related to maternal nutritional conditions. In this context, the rising incidence of arterial hypertension, diabetes type II, and dyslipidemia has been attributed to genetic programming. Besides, environmental conditions during perinatal development such as maternal undernutrition or overnutrition can program changes in the integration among physiological systems leading to cardiometabolic diseases. This phenomenon can be understood in the context of the phenotypic plasticity and refers to the adjustment of a phenotype in response to environmental input without genetic change, following a novel, or unusual input during development. Experimental studies indicate that fetal exposure to an adverse maternal environment may alter the morphology and physiology that contribute to the development of cardiometabolic diseases. It has been shown that both maternal protein restriction and overnutrition alter the central and peripheral control of arterial pressure and metabolism. This review will address the new concepts on the maternal diet induced-cardiometabolic diseases that include the potential role of the perinatal malnutrition.

## Introduction

Cardiovascular and metabolic diseases, such as hypertension, type II diabetes, and dyslipidemia are highly prevalent in the world and have important effects on the public health, increasing risk factors for the development of other diseases, including coronary heart disease, stroke, and heart failure (Landsberg et al., 2013). The etiology of these cardiometabolic diseases includes a complex phenotype that arises from numerous genetic, environmental, nutritional, behavioral, and ethnic origins (Landsberg et al., 2013; Ng et al., 2014). In this regard, it has been observed that the eating habits and behaviors and nutritional condition in early phases of life may play a key role on the etiology of these diseases by inducing physiological dysfunctions (Lucas, 1998; Victora et al., 2008; Wells, 2012). This phenomenon can be understood in the context of phenotypic plasticity and it refers to the ability of an organism to react to both an internal and external environmental inputs with a change in the form, state, physiology, or rate of activity without genetic changes (West-Eberhard, 2005b). Indeed the nutritional factors rise as important element in this theme and it has been highlighted since Barker (Barker, 1990, 1994, 1995, 1998, 1999a,b, 2000; Barker and Martyn, 1992; Fall and Barker, 1997; Osmond and Barker, 2000). In this context, new evidence from epidemiological and clinical studies have showed the association of the maternal under- and overnutrition with development of cardiometabolic dysfuntions (Ashton, 2000; Hemachandra et al., 2006; Antony and Laxmaiah, 2008; Conde and Monteiro, 2014; Costa-Silva et al., 2015; Parra et al., 2015). Thus, this review will address the new concepts about the involvement of the maternal protein malnutrition and overnutrition on the development of the cardiometabolic diseases.

## Perinatal origin of cardiometabolic diseases: the role of phenotypic plasticity

Biological and medical consequences of perinatal nutritional factors have been extensively studied in the field of the “developmental origins of health and diseases” proposed by Barker and colleagues since 1986 (Barker and Osmond, 1986; Barker et al., 1989, 1993; Barker, 2007). This field of research proposes that cardiometabolic diseases can be “programmed” by the “adaptative” effects of both under- and overnutrition during early phases of growth and development on the cell physiology (Barker and Osmond, 1986; Hales and Barker, 1992; Alfaradhi and Ozanne, 2011; Chavatte-Palmer et al., 2016). As stated before, it aims to study how an organism reacts to a different environmental input, such as malnutrition, and induces changes in the phenotype, but without altering the genotype (Barker et al., 2005; West-Eberhard, 2005a; Labayen et al., 2006; Andersen et al., 2009; Biosca et al., 2011). In this context, epigenetic alterations, such as DNA methylation, histone acetylation, and microRNA expression are considered the molecular basis of the phenotypic plasticity (Wells, 2011). These modifications termed as “epigenetic” were firstly described by Conrad Waddigton in 1940 and it studies the relationship between cause and effect in the genes to produce a phenotype (Jablonka and Lamb, 2002). Nowadays, this concept is employed to describe the process of the gene expression and its linking to modifications in the cromatin structure without altering DNA sequence (Chong and Whitelaw, 2004; Egger et al., 2004). Among all epigenetic modifications, the DNA methylation is one that has been best studied and is related to addition of methyl groups on DNA cytosine residues, normally on the cytosine followed by guanine residue (CpG dinucleotides), which can produce inhibition of the gene expression by impairing transcriptional factor binding (Waterland and Michels, 2007; Mansego et al., 2013; Chango and Pogribny, 2015; Mitchell et al., 2016). In this context, it has been investigated how nutritional aspect may induce these epigenetic modifications.

Macro- and micro-nutrient compositions have been identified as important nutritional factors inducing epigenetic processes, such as DNA methylation (Mazzio and Soliman, 2014; Szarc vel Szic et al., 2015). It is considered at least three ways by which nutrients can induce DNA methylation, alter gene expression, and modify cellular phenotype: (i) by providing methyl group supply for inducing S- adenosyl-L-methionine formation (genomic DNA methylation), modifying the methyltransferase activity, or impairing DNA demethylation process; (ii) by modifying chromatin remodeling, or lysine and arginine residues in the N-terminal histone tails; and (iii) by altering microRNA expression (Chong and Whitelaw, 2004; Egger et al., 2004; Hardy and Tollefsbol, 2011; Stone et al., 2011). In this context, altered contents of amino acids, such as methionine and cysteine, as well as reduced choline and folate diet amount can modify the process of the DNA methylation leading to both DNA hyper- and hypomethylation (Fiorito et al., 2014). For example, deficiency of choline can precipitate DNA hypermethylation associated with organ dysfunction, mainly in liver metabolism (Karlic and Varga, 2011; Wei, 2013).

High fat diet (HFD) during perinatal period has been identified as risk factor to predispose and induce epigenetic processes in the parents and their offspring (Mazzio and Soliman, 2014; Szarc vel Szic et al., 2015). Both hypo- and hypermethylation processes participate in this dysregulation attributed to HFD consumption (Ng et al., 2010; Milagro et al., 2013). In adipose tissue, for example, it was observed that gene promoter of the fatty acid synthase enzyme suffered methylation (Lomba et al., 2010) and that important obesity-related genes such as leptin have disruption on their methylation status (Milagro et al., 2009).

## Maternal protein undernutrition: early- and long-term outcomes

Maternal malnutrition is associated with the risk of developing cardiovascular disease and co-morbidities in offspring's later life including hypertension, metabolic syndrome, and type-II diabetes (Barker et al., 2007; Nuyt, 2008; Nuyt and Alexander, 2009). In humans, studies have provided support for the positive association between low birth weight and increased incidence of hypertension (Ravelli et al., 1976; Hales et al., 1991; Sawaya and Roberts, 2003; Sawaya et al., 2004).

Maternal low-protein diet model during both gestation and lactation is one of the most extensively studied animal models of phenotypic plasticity (Ozanne and Hales, 2004; Costa-Silva et al., 2009; Falcão-Tebas et al., 2012; Fidalgo et al., 2013; de Brito Alves et al., 2014; Barros et al., 2015). Feeding a low-protein diet (8% protein) during gestation and lactation is associated with growth restriction, asymmetric reduction in organ growth, elevated systolic blood pressure, dyslipidemia, and increased fasting plasma insulin concentrations in the most of studies in rodents (Ozanne and Hales, 2004; Costa-Silva et al., 2009; Falcão-Tebas et al., 2012; Fidalgo et al., 2013; Leandro et al., 2012; de Brito Alves et al., 2014, 2016; Ferreira et al., 2015; Paulino-Silva and Costa-Silva, 2016). However, it is known that the magnitude of the cardiovascular and metabolic outcomes are dependent on the both time exposure to protein restricted-diet (Zohdi et al., 2012, 2015) and growth trajectory throughout the postnatal period (Wells, 2007, 2011). A rapid and increased catch-up growth and childhood weight gain appear to augment metabolic disruption in end organs, for example liver (Tarry-Adkins et al., 2016; Wang et al., 2016).

Although, the relationship between maternal protein restriction, sympathetic overactivity and hypertension have been suggested (Johansson et al., 2007; Franco et al., 2008; Barros et al., 2015), few studies have described the physiological dysfunctions responsible for producing these effects. Nowadays, it is well accepted that perinatal protein malnutrition raise risks of hypertension by mechanisms that include abnormal vascular function (Franco Mdo et al., 2002; Brawley et al., 2003; Franco et al., 2008), altered nephron morphology and function, and stimulation of the renin-angiotensin system (RAS) (Nuyt and Alexander, 2009; Siddique et al., 2014). Recently, studies have highlighted contribution of the sympathetic overactivity associated to enhanced respiratory rhythm and O_2_/CO_2_ sensitivity on the development of the maternal low-protein diet-induced hypertension by mechanisms independent of the baroreflex function (Chen et al., 2010; Barros et al., 2015; Costa-Silva et al., 2015; de Brito Alves et al., 2015; Paulino-Silva and Costa-Silva, 2016). Offspring from dams subjected to perinatal protein restriction had relevant short-term effects on the carotid body (CB) sensitivity and respiratory control. With enhanced baseline sympathetic activity and amplified ventilatory and sympathetic responses to peripheral chemoreflex activation, prior to the establishment of hypertension (de Brito Alves et al., 2014, 2015). The underlying mechanism involved in these effects seems to be linked with up-regulation of hypoxic inducible factor (HIF-1α) in CB peripheral chemoreceptors (Ito et al., 2011, 2012; de Brito Alves et al., 2015). However, the epigenetic mechanisms in these effects are still unclear. It is hypothesized that epigenetic mechanism produced by DNA methylation could be involved (Altobelli et al., 2013; Prabhakar, 2013; Nanduri and Prabhakar, 2015).

The central nervous system (CNS) compared to other organ systems has increased vulnerability to reactive oxygen species (ROS). ROS are known to modulate the sympathetic activity and their increased production in key brainstem sites is involved in the etiology of several cardiovascular diseases, for example, diseases caused by sympathetic overexcitation, such as neurogenic hypertension (Chan et al., 2006; Essick and Sam, 2010). Ferreira and colleagues showed that perinatal protein undernutrition increased lipid peroxidation and decreased the activity of several antioxidant enzymes (superoxide dismutase, catalase, glutathione peroxidase, and glutathione reductase activities) as well as elements of the GSH system, in adult brainstem. Dysfunction in the brainstem oxidative metabolism, using the same experimental model, were observed in rats immediately after weaning associated to the increase in ROS production, with a decrease in antioxidant defense and redox status (Ferreira et al., 2015, 2016). Related to the metabolic effects on the heart, it was observed that these animals showed decreased mitochondrial oxidative phosphorylation capacity and increased ROS in the myocardium. In addition, maternal low-protein diet induced a significant decrease in enzymatic antioxidant capacity (superoxide dismutase, catalase, glutathione-S-transferase, and glutathione reductase activities) and glutathione level when compared with normoprotein group (Nascimento et al., 2014).

Regarding hepatic metabolism, studies showed that protein restricted rats had suppressed gluconeogenesis by a mechanism primarily mediated by decrease on the mRNA level of hepatic phosphoenolpyruvate carboxykinase, a key gluconeogenic enzyme, and enhancement of the insulin signals through the insulin receptor (IR)/IR substrate (IRS)/phosphatidylinositol 3-kinase (PI3K)/mammalian target of rapamycin complex 1 (mTOR) pathway in the liver (Toyoshima et al., 2010). In relation to lipid metabolism, there was decreased liver triglyceride content in adult rats exposed to protein restriction during gestation and lactation. It was suggested that this effect could be due to increased fatty-acid transport into the mitochondrial matrix or alterations in triglyceride biosynthesis (Qasem et al., 2015). A maternal protein restriction was shown to reduce the lean and increase the fat contents of 6-month old offspring with a tendency for reduced number of muscle myofibers associated with reduced expression of mRNA of Insulin-like growth factor 2 gene (IGF2 mRNA) in pigs (Chavatte-Palmer et al., 2016).

## Maternal overnutrition and risk factor for the cardiometabolic dysfuntions

Nutritional transition is a phenomenon well documented in developing countries in the twentieth and twenty-first centuries, and has induced high incidence of the chronic diseases and high prevalence of the obesity (Batista Filho and Rissin, 2003; Batista Filho and Batista, 2010; Ribeiro et al., 2015). It is evident that protein malnutrition was an health problem in the first half of the twentieth century. Now, it was replaced by a diet enriched in saturated fat or other HFDs, predisposing to overweight, and obesity (Batista et al., 2013). Nowadays, it suggested that two billion people in the world are overweight and obese individuals, with major prevalence is related to diet induced-obesity, which have been associated to cardiovascular and endocrine dysfunctions (Hotamisligil, 2006; Aubin et al., 2008; Zhang et al., 2012; Ng et al., 2014; Wensveen et al., 2015).

Recently, the obesity has been considered a physiological state of chronic inflammation, characterized by elevated levels of inflammatory markers including C-reactive protein (CRP), interleukin-6 (IL-6), and tumor necrosis factor alpha (TNF-α) (Wensveen et al., 2015; Erikci Ertunc and Hotamisligil, 2016; Lyons et al., 2016). Maternal HFD chronic consumption enhances the circulating free fatty acids and induce the activation of inflammatory pathways, enhancing chronic inflammation in offspring (Gruber et al., 2015). Studies of Roberts et al. (2015) found that cardiometabolic dysfunction was associated with changes such as elevated serum triglycerides, elevated oxidative stress levels, insulin resistance, vascular disorders, and development of hypertension (Roberts et al., 2015).

In animals on a HFD the hormone leptin has been considered one of the most important physiological mediators of the cardiometabolic dysfunction (Correia and Rahmouni, 2006; Harlan et al., 2013; Harlan and Rahmouni, 2013). Since hyperleptinemia, common in overweight and obesity conditions, produce a misbalance in autonomic system, with sympathetic overactivation (Machleidt et al., 2013; Kurajoh et al., 2015; Manna and Jain, 2015), and reduced sensitivity of vagal afferent neurons (de Lartigue, 2016). This disorder of vagal afferent signaling can activate orexigenic pathways in the CNS and drive hyperphagia, obesity, and cardiometabolic diseases at long-term (de Lartigue, 2016). Some authors have described that, at least in part, cardiovascular dysfuntion elicited by HFD or obesity may be due to changes in the neural control of respiratory and autonomic systems (Bassi et al., 2012, 2015; Hall et al., 2015; Chaar et al., 2016). Part of these effects were suggested to be influenced by atrial natriuretric peptide and renin-angiotensin pathways (Bassi et al., 2012; Gusmão, 2012).

Interestingly, it has been shown that offspring from mothers fed HFD have high risk to develop pathologic cardiac hypertrophy. This condition would be linked to re-expression of cardiac fetal genes, systolic, and diastolic dysfunction and sympathetic overactivity on the heart. These effects lead to reduced cardioprotective signaling that would predispose them to cardiac dysfunctions in adulthood (Taylor et al., 2005; Wang et al., 2010; Fernandez-Twinn et al., 2012; Blackmore et al., 2014). Regarding arterial blood pressure control, it has been described that maternal HFD induces early and persistent alterations in offspring renal and adipose RAS components (Armitage et al., 2005). These changes seem to be dependent upon the period of exposure to the maternal HFD, and contribute to increased adiposity and hypertension in offspring (Samuelsson et al., 2008; Elahi et al., 2009; Guberman et al., 2013; Mazzio and Soliman, 2014; Tan et al., 2015). Studies in baboons subjected to HFD showed that microRNA expression and putative gene targets involved in developmental disorders and cardiovascular diseases were up-regulated and others were down-regulated. The authors suggested that the epigenetic modifications caused by HFD may be involved in the developmental origins of cardiometabolic diseases (Maloyan et al., 2013).

Other metabolic outcomes induced by HFD have been pointed out in the last years and it has demonstrated that HFD displayed a drastic modification on metabolic control of the glucose metabolism and lead to increased insulin level in serum (Fan et al., 2013) and enhanced insulin action through AKT/PKB (protein kinase B) and ERK (extracellular signal-regulated kinase), and activation of mammalian target of rapamycin (mTOR) pathways in cardiac tissue (Fernandez-Twinn et al., 2012; Fan et al., 2013). Offspring from HFD mothers showed alterations in blood glucose and insulin levels, with high predisposition to insulin resistance and cardiac dysfunction (Taylor et al., 2005; Wang et al., 2010). Part of these effects are associated with enhanced production of ROS and reduction in the levels of the anti-oxidant enzymes, such as superoxide dismutase, suggesting a misbalance in the control of the oxidative stress (Fernandez-Twinn et al., 2012).

**Figure 1:**
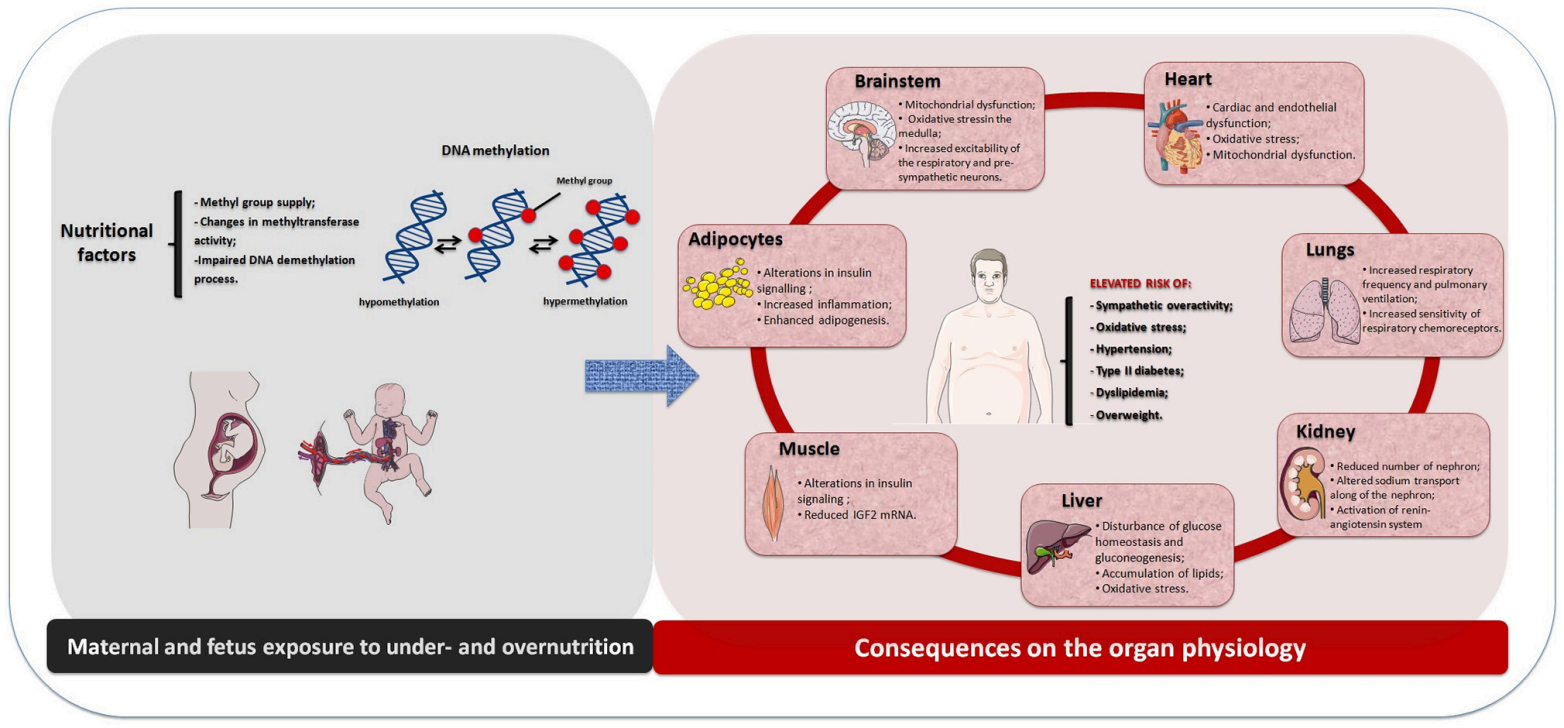
**Schematic drawing showing the physiological effects induced by maternal and fetus exposure to under- or overnutrition through DNA methylation and their consequences on the organ physiology and increased risk of the cardiometabolic diseases in the offspring**.

Altogether, this review addressed the new concept on the maternal diet induced-cardiometabolic diseases that include the potential role of the perinatal malnutrition. It showed that the etiology of these diseases is multifactorial involving genetic and environmental influences and their physiological integration. It is well recognized that both perinatal undernutrition and overnutrition are related with the risk of developing metabolic syndrome and hypertension in adult life (Figure 1). The underlying mechanism can be explained in the context of phenotypic plasticity during development that includes adaptive change on the CNS, heart, kidney, liver, muscle, and adipose tissue metabolisms with consequent physiology dysfunction and with subsequent cardiometabolic diseases. Moreover, maternal undernutrition or overnutrition may predispose epigenetic modifications in dams and their offspring, with predominance of DNA methylation, leading to altered gene expression during development and growth. Further, it can provide a different physiological condition which may contribute to the developmental origins of the cardiometabolic diseases. These physiological dysfunctions seem to be linked to the impaired central and peripheral control of both metabolic and cardiovascular functions by mechanisms that include enhanced sympathetic-respiratory activities and disruption in metabolism of end organs at early life. It is suggested that those effects could be associated to inflammatory conditions and impaired oxidative balance, which may contribute to adult cardiometabolic diseases.

## Author contributions

JC, AS, and MF drafted and revised critically the work for important intellectual content and final review of the manuscript.

### Conflict of interest statement

The authors declare that the research was conducted in the absence of any commercial or financial relationships that could be construed as a potential conflict of interest.

## Abbreviations

AKT/PKB: Protein kinase B
CB: Carotid body
CNS: Central nervous system
CRP: C-reactive protein
ERK: Extracellular signal-regulated kinase
GSH: Glutathione reduced
HFD: High fat diet
HIF-1α: Hypoxic inducible factor 1 alpha
IGF2: Insulin-like growth factor 2
IL-6: Interleukin-6
IR: Insulin receptor
IRS: Insulin receptor substrate
mTOR: Mammalian target of rapamycin
PI3K: Phosphatidylinositol 3-kinase
RAS: Renin-angiotensin system
ROS: Reactive oxygen species
TNF-α: Tumor necrosis factor alpha.
